# Supplementary material for: Dinuclear Iron Complexes of Iminopyridine-Based Ligands as Selective Cytotoxins for Tumor Cells and Inhibitors of Cancer Cell Migration
Source: Pharmaceutics. 2022 Dec 14;14(12):2801. doi: 10.3390/pharmaceutics14122801 (PMC9781652; doi:10.3390/pharmaceutics14122801)
Supplement: Supplementary file 1 [file pharmaceutics-14-02801-s001.zip › pharmaceutics-1953187-supplementary.pdf]

Supplementary Materials: Dinuclear Iron Complexes of Iminopyridine-Based Ligands as Selective Cytotoxins for Tumor Cells and Inhibitors of Cancer Cell Migration

Jessica Castro, Marlon Bravo, Meritxell Albertí, Anaís Marsal, María José Alonso-De Gennaro, Oriol Martínez-Ferraté, Carmen Claver, Piet W. N. M. van Leeuwen, Isabel Romero, Antoni Benito and Maria Vilanova

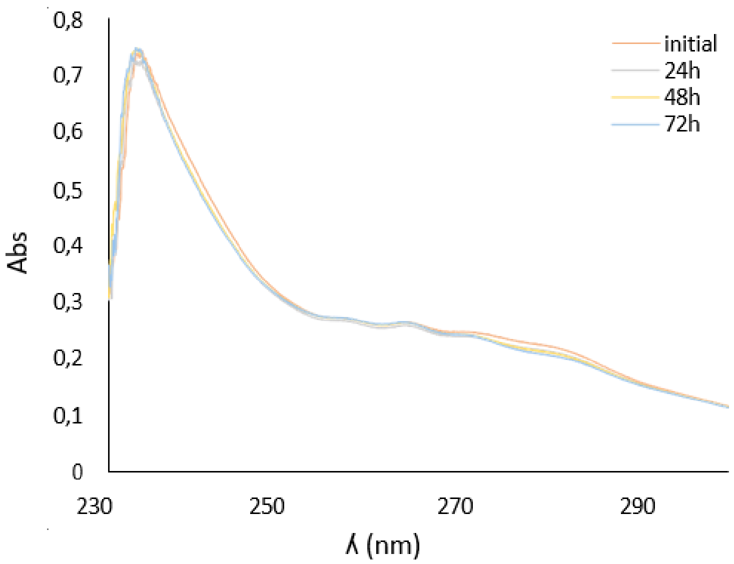

(**A**)

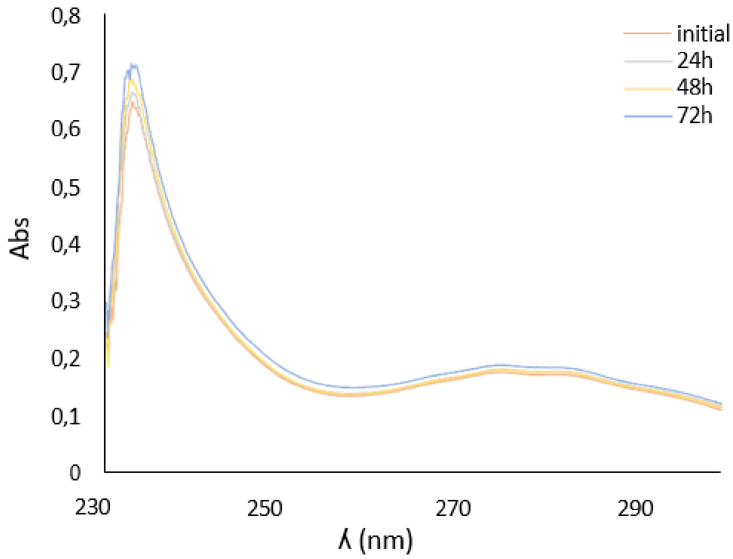

(**B**)

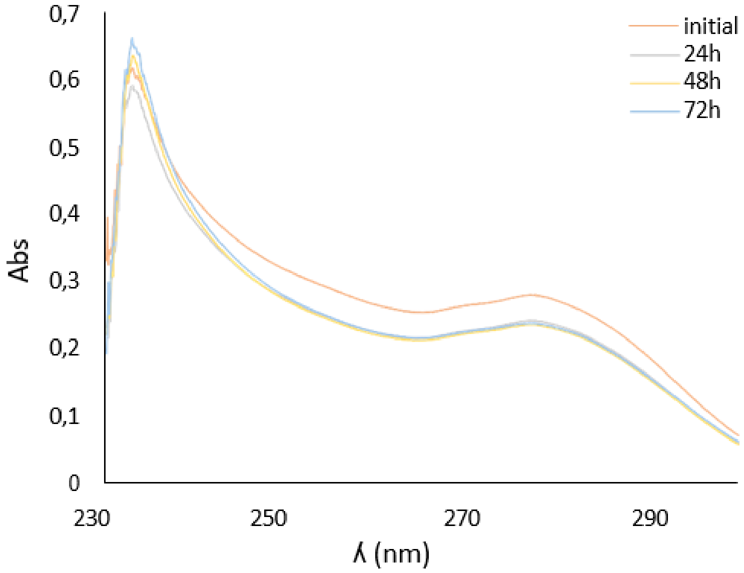

0,7
0,6
0,5
0,4
0,3
0,2
0,1
0
Abs
230
250
270
290
ʎ (nm)
initial
24h
48h
72h

(C)

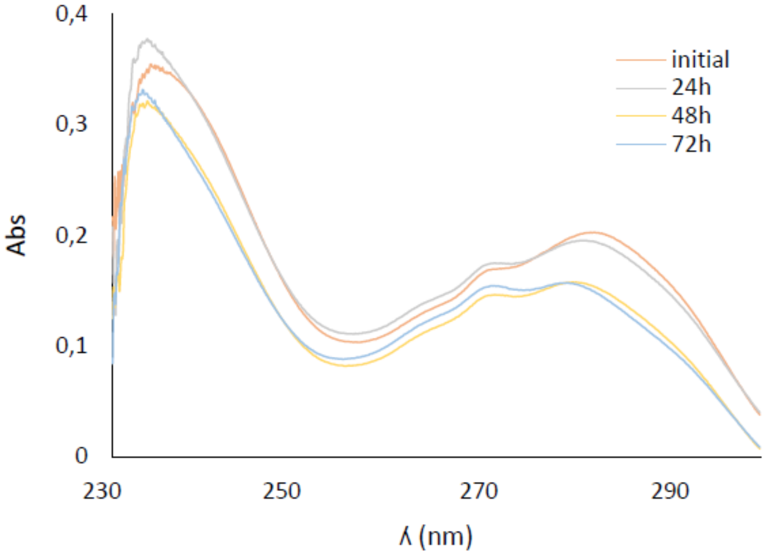

0,4
0,3
0,2
0,1
0
Abs
230
250
270
290
ʎ (nm)
initial
24h
48h
72h

(D)

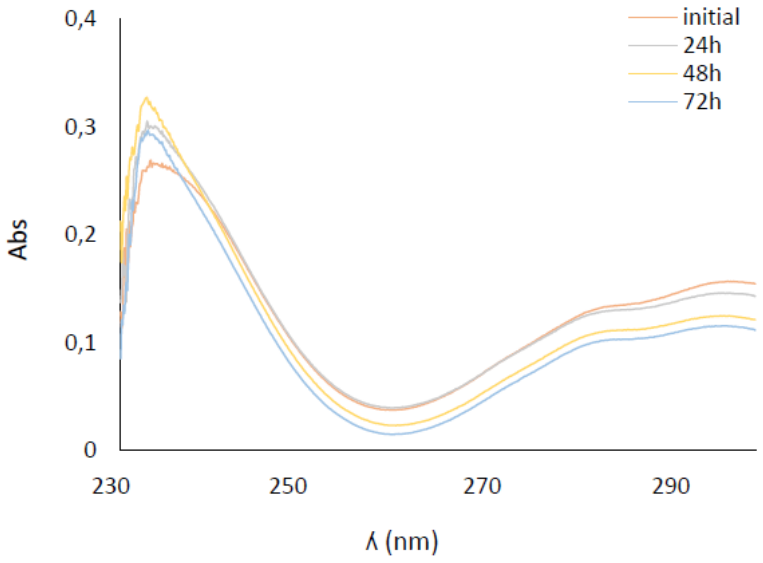

(**E**)

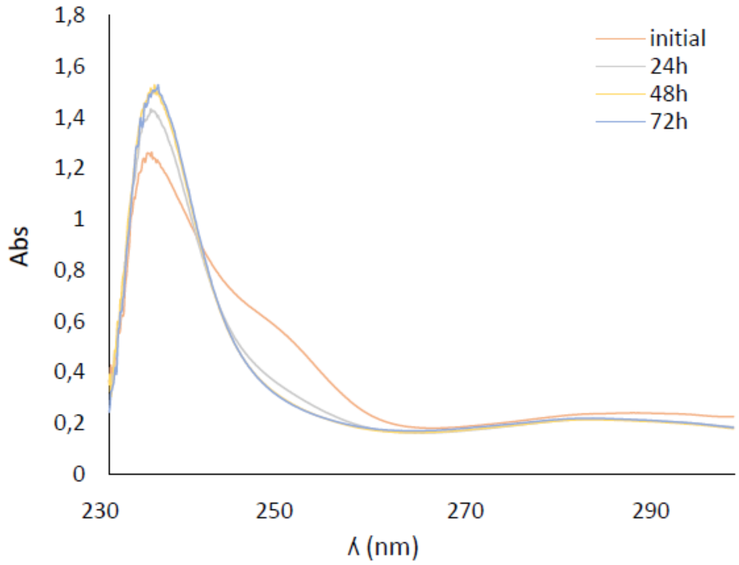

(**F**)

Figure S1. Stability of complexes. The stability of compounds **7** (**A**), **8** (**B**), **9** (**C**), **10** (**D**), **11** (E) and **12** (**F**) has been studied in physiological conditions for up to 72 h. UV-spectra registered along the time for all the assayed compounds are displayed.
